# Pharmacogénétique: qu'en est-il au Maroc?

**DOI:** 10.11604/pamj.2013.14.143.2141

**Published:** 2013-04-10

**Authors:** Meryem Janati Idrissi, Karim Ouldim, Afaf Amarti, Mohamed El Hassouni, Youssef Khabbal

**Affiliations:** 1 Laboratoire Central des Analyses Médicales, CHU Hassan II, Fès; 2 Faculté de Médecine et de Pharmacie, Université Sidi Mohamed Ben Abdellah, Fès; 3 Laboratoire de Biotechnologie, Faculté des sciences Dhar El Mahraz, Université Sidi Mohamed Ben Abdellah, Fès, Maroc

**Keywords:** Pharmacogénétique, Maroc, médicine personnalisée, métabolisme, médicaments, pharmacogenetics, Morocco, personalized medicine, metabolism, drugs

## Abstract

La pharmacogénétique est l’étude de l'influence des variations génétiques interindividuelles sur la réponse aux médicaments, avec le but d'améliorer la prise en charge des patients en visant une médecine personnalisée. Au fait le génome de deux personnes ne diffère que par 0.1% des 3.2 milliards de paires de bases, ce qui implique les effets indésirables des médicaments, qui ont un très important impact sur le plan clinique que sur le plan économique. Or cette dernière décennie ces effets indésirables ont pu être évités grâce aux tests pharmacogénétiques. Au Maroc, la recherche en pharmacogénétique commence à susciter l'intérêt des chercheurs avec quelques études. Une toute première étude en 1986, sur l'acétylation de l'isoniazide chez la population marocaine, suivie par deux autres en 2011 se focalisant sur le métabolisme du tacrolimus et des anti-vitamines K. Ainsi l'espoir maintenant est d'identifier les majoritaires polymorphismes génétiques affectant les patients marocains, afin de leur fournir une prise en charge adaptée.

## Introduction

La réponse aux médicaments est extrêmement variable d'un individu à l'autre, tant sur le plan pharmacologique (efficacité) que sur le plan toxicologique (effets indésirables). La variabilité de cette réponse, souvent difficile à prévoir, est une limitation importante à l'utilisation des médicaments [1]. Dans le cas de ceux ayant une fenêtre thérapeutique étroite, la prédiction de l'efficacité et de la toxicité devient un problème crucial. Outre la marge étroite, des polymorphismes génétiques peuvent influencer la réponse pharmacologique des médicaments, ces polymorphismes intéressent surtout les transporteurs et les enzymes impliquées dans le métabolisme. La pharmacogénétique se définit selon l'European Medecine agency (EMEA) comme étant l'influence des variations interindividuelles de la séquence de l'ADN sur la réponse à l'effet des médicaments. Cette approche vise à développer des tests simples de dépistage de polymorphisme génétique permettant d'identifier les individus exposés aux effets indésirables des traitements médicamenteux. Dans cette revue, quelques principes dont se base la pharmacogénétique sont exposés; avec l’évocation des enjeux économiques et cliniques de cette discipline, en donnant un aperçu sur la situation du Maroc par rapport à la pharmacogénétique.

## Méthodes

Une première recherche sur PubMed et sur ScienceDirect en utilisant les mots clés “pharmacogenetics”, “personalized medecine” et “drug responce” a permis d′obtenir une vision globale sur la découverte et le développement de cette discipline, ainsi que son intérêt clinique. La deuxième recherche est faite en utilisant les mots suivants: “cytochrome 2D6”, “thiopurine S-methyltransferase”, “SLCO1B1” et “vitamine K-époxyde réductase”, qui représentent quarte exemples pertinents des enjeux de la pharmacogénétique en pratique médicale. Les articles les plus pertinents sont retenus.

## État actuel des connaissances

### A quoi est due la variabilité interindividuelle?

La variabilité de la pharmacocinétique d'une molécule entre deux individus peut dépendre de différents facteurs: le polymorphisme génétique, les facteurs environnementaux (alimentation, co-administration de médicaments ou tabagisme), le statut physiologique et l'existence de maladies intercurrentes.

Le facteur génétique et le facteur environnement sont les deux causes principales dans la survenue d'effets indésirables ou d'inefficacité de traitement [2]. Étudier le polymorphisme génétique consiste à étudier soit les variations du phénotype protéinique, soit étudier directement l'ADN. Le génome ne change pas avec le temps, ce qui fait de lui un objet d’étude invariant pour un individu donné; contrairement au phénotype qui en plus d’être le résultat de l'expression des gènes, il est sujet à d'autres influences des facteurs environnementaux.

Le génotype de deux personnes ne diffère que par un faible pourcentage: seuls 0,1% des 3,2 milliards de paires de bases constituant le génome de deux humains suffisent à les différencier. Les différences les plus souvent observées ne dépendent que d'une seule base azotée (substitution, délétion, addition), on parle dans ce cas de “Single Nucleotide Polymorphism” (SNP). Le nombre de SNP entre deux humains est de l'ordre de 2.5 millions (1 pour 1250 paires de bases) dont la majorité est répertoriée et classée. Les SNP peuvent survenir dans différentes régions du génome: certains sont situés sur les régions codantes de l'ADN (coding SNPs), d'autres sont situés sur les introns (perigenic SNPs) et d'autres se trouvent dans les régions intergéniques (intergenic SNPs). Bien entendu, tous n'entraînent pas de variations phénotypiques (la majorité est d'ailleurs silencieuse).

### Intérêt de la pharmacogénétique

#### A. Enjeux cliniques

La pharmacogénétique est la discipline s'intéressant aux réponses pharmacologiques et de leurs modifications d'origine génétique [3]. Dans le but d'améliorer la prise en charge des patients, en se basant sur le concept de ‘la bonne dose au bon patient’ [4], Cette approche de la médecine personnalisée vise aux traitements thérapeutiques sur mesure, en prenant en considération les profils clinicobiologique et génétique de chaque patient, réduisant ainsi le risque de survenu de toxicité due aux médicaments. Les SNP particulièrement intéressants sont ceux dont la mutation entraîne une modification fonctionnelle ou structurale de la protéine correspondante. On estime 200 à 300, le nombre des processus pharmacologiques reconnus comme étant sujets à un polymorphisme génétique. Pour s'y retrouver, les polymorphismes sont classés selon l’étape du métabolisme affectée.

**1. Phase I du métabolisme:** La plupart des polymorphismes identifiés et mieux étudiés à ce jour, sont les cytochromes P450 impliqués dans l'oxydation des médicaments (CYP 1A2, 2B6, 2C9, 2C19, 2D6, 3A4, 3A5). Le déficit de ces enzymes incite un ralentissement dans l’élimination des médicaments, ou une absorption accrue par diminution du premier passage hépatique. Par contre les pro-médicaments nécessitant le métabolisme pour leur bio-activation voient leur efficacité diminuer. Le CYP 2D6 métabolisant environ 25% des médicaments (psychotropes, anticancéreux), est le premier cytochrome pour lequel un polymorphisme a été identifié dans les années 1970 [5]. Les différentes mutations affectant le CYP2D6 expliquent la survenue de toxicités et de non-réponse à de nombreux médicaments de cette voie métabolique. Cependant la capacité prédictive d'un génotypage, peut être confondue par la prescription de médicaments capables d'inhiber ou de stimuler ce cytochrome. Le tamoxifène, un anti-‘strogène prescrit en prévention secondaire dans le cancer du sein, subit une déméthylation et une hydroxylation donnant naissance à deux métabolites puissants, responsables de la majeure partie de l'activité thérapeutique. Or chez les métaboliseurs lents du CYP2D6 et chez les individus recevant un inhibiteur de cette voie, cette transformation ne peut avoir lieu [6, 7]. Le gène codant pour cette enzyme hépatique se localisé sur le chromosome 22q13, et il est très polymorphe avec plus de 71 polymorphismes identifiés (http://www.cypalleles.ki.se/cyp2d6.htm), dont les plus importants et qui permettent une prédiction de 90% sont: CYP2D6 *;3, CYP2D6*;4, CYP2D6 *;5 (délétion du gène entier) et CYP2D6 *;6 (duplication du gène variante de 2 à 13 fois).

**2. Phase II du métabolisme:** Le déficit des transférases (N-acétyltransférase, thiopurine S-methyltransférase, UDP-glucuronyltransférase) engendre un ralentissement dans l’élimination des médicaments, ou bien une accumulation de métabolites secondaires actifs. La thiopurine S-methyltransférase (TPMT) assure la méthylation des médicaments thiopurines (Azathioprine et 6-mercaptopurine) ainsi que leur métabolite actif, ce qui les désactives; c'est une enzyme cytoplasmique présente dans de nombreux tissus (cellules sanguines, cœur, intestin). Chez l'Homme, le gène localisé sur le chromosome 6p22 comprend dix exons dont huit sont codants; et il est très polymorphe avec environ 26 allèles reportés (selon la Base de pharmGKB). Au moins 4 polymorphismes sont connus d’être responsable d'un phénotype déficient par une activité enzymatique réduite, voire nulle, ce qui conduit à une augmentation des taux plasmatiques du métabolite actif toxique [8, 9]. Ces polymorphismes déterminent quatre allèles mutés TPMT*2, TPMT*3A, TPMT*3B et TPMT*3C dont la détermination permet la prédiction du phénotype de cette enzyme avec une efficacité d'environ de 90 à 95% [10].

**3. Transport:** L'importance des transporteurs transmembranaires de métabolites ayant une activité pharmacologique est de mieux en mieux reconnue. Lorsqu'un polymorphisme affecte les transporteurs tels que la P-glycoprotéine et divers analogues, on obtient des transporteurs dits lents, qui affectent la pharmacocinétique des médicaments ou leur pénétration dans les cellules saines ou tumorales (cas des anticancéreux) [11]. Le transporteur membranaire SLCOC1B1 appartient à la famille des transporteurs SLCO Solute carrier organic anion transporter family, connue aussi sous le nom Organic anion transporting polypeptides (OATP). Cette famille regroupe les transporteurs membranaires soutenant l'influx cellulaire d'une variété de médicaments. Le SLCO1B1 est exprimé dans la membrane sinusoïdale des hépatocytes, et entraine le passage d'un grand nombre de médicaments tels que les statines. Ce passage vers les cellules hépatiques ne représente pas seulement la première étape de l’élimination hépatique de ces médicaments, mais aussi un système de délivrance vers le foie, qui est l'organe cible [12]. Le transport peut influencer l'efficacité thérapeutique des statines, puisque leur niveau plasmatique varie selon l'activité du transporteur, ce qui cause la variabilité de la réponse thérapeutique. Le gène codant pour le transporteur SLCO1B1 se localise sur le chromosome 12p12, et plusieurs polymorphismes affectent ce gène, dont deux sont incriminés dans l'augmentation des taux plasmatiques des statines [12], et qui sont: SLCO1B1*5 (T521C) et SLCO1B1*1b (A388G).

**4. Récepteurs:** La pharmacodynamie des médicaments est affectée aussi par les polymorphismes génétiques; dont plusieurs exemples sont connus tels que l'enzyme de conversion de l'angiotensine (réponse aux antidépresseurs) et la vitamine K-époxyde réductase. La vitamine K-époxyde réductase (VKORC1) est la cible pharmacologique des médicaments antivitamines K (AVK). Ces médicaments inhibent le cycle de régénération de la vitamine K, par blocage de la réduction de la vitamine K époxyde (KO) en viatmine K sous forme quinone (K) par l'inhibition compétitive de VKORC1 au niveau des sites d'activation enzymatique de l’époxyde réductase [13]. Le gène de VKORC1 se situe sur le chromosome 16, il comprend 3 exons et s’étend sur 5 Kb. Il code pour une réductase di-thiol-dépendante qui transforme la vitamine K époxyde en vitamine K quinone [14].

#### B. Enjeux économiques

L'iatrogénie médicamenteuse est responsable d'environ 7% des hospitalisations [15], d'un peu près de 20% des réadmissions à l'hôpital [16], et de 4% des retirements du marché de nouvelles molécules [17]. Chaque année en France, les effets indésirables des médicaments sont responsables d'environ 128 000 hospitalisations avec une incidence de 3.2%, pour un coût global estimé à 320 millions d'euros (afssaps 2001). Ces chiffres démontrent que les variations individuelles de réponse aux médicaments représentent un problème médical et de Santé Publique important. Aux Etats-Unis, 6.7% des hospitalisations, dont 100.000 décès sont dus aux effets indésirables des médicaments [18, 19].

### Qu'en est-il au Maroc?

Au Maroc, le domaine de la recherche médicale reste presque désertique malgré le grand intérêt qu'il représente pour les cliniciens, dans le cadre de l'amélioration des soins. Cette stagnation dans la recherche médicale commence à se dissiper; ce qui conduit à de nouvelles approches pour la prise en charge des malades. Ces dernières années, la pharmacogénétique commence à susciter l'attention des chercheurs scientifiques et des cliniciens, vu qu'elle évoque l'espoir d'une prise en charge plus optimisée des patients. Bien que la première étude dans ce domaine fut en 1982 [20], par une étude sur l'acétylation de l'isoniazide qui a été publiée par Bouayad et al. L’étude avait pour objectif de déterminer le phénotype de 100 patients vis-à-vis l'isoniazide. Les résultats ont montré que la majorité était des métaboliseurs rapides, ce qui explique la bonne tolérance de la dose administrée (10 mg/Kg). Mais depuis il n'y a eu aucune étude publiée dans ce sens. Ce n'est qu'en 2011 que de nouvelles études ont émergé. La première étude se focalise sur l'effet des variantes génétiques du cytochrome 2C9 (CYP2C9) et de la vitamine K peroxydase (VKORC1) sur la sensibilité des patients marocains vis-à-vis l'acénocoumarol. L’évaluation des fréquences alléliques de CYP2C9*2, de CYP2C9*3 et de VKORC1 ‘1639 G > A montre que ces allèles modulent la sensibilité à l'acénocoumarol, d'où la nécessité de tests prédictifs concernant ces polymorphismes avant chaque prescription [21]. La deuxième étude se porte sur le cytochrome 3A5 (CYP3A5) avec la détermination de la fréquence des allèles CYP3A5*1 et CYP3A5*3 chez la population marocaine et l’évaluation de leur effet sur la dose journalière du Tacrolimus pour les patients avec transplantation rénale [22]. Ces études sont les premières à fournir les données génétiques en relation avec la fréquence des polymorphismes de CYP2C9, CYP3A5 et VKORC1 dans la population marocaine, ce qui ouvre la perspective du développement de nouvelles études pharmacogénétiques.

### Pharmacogénétique et nouvelles technologies

L'impact du génome humain sur la prédiction de la réponse aux traitements médicamenteux, est la base de la pharmacogénétique, dont le développement oriente vers une médecine personnalisée des patients selon leurs profils génétiques. La pharmacogénétique utilise les technologies génomiques pour comprendre les effets de tous les gènes ayant une implication dans le métabolisme des médicaments, et ces dernières années plusieurs tests pharmacogénétiques sont reconnus par les organisations réglementaires et scientifiques, d'avoir un potentiel de moduler la pratique clinique. Les techniques d'analyse de l'ADN doivent être simples, rapides et particulièrement fiables, lors de la pratique clinique courante. Dans la pharmacogénétique, l'analyse du gène affectant le métabolisme d'un médicament, commence par l'amplification de ce gène, ou bien d'un fragment par la réaction de polymérisation en chaine (PCR) puis son analyse s'effectue par électrophorèse ou par séquençage. Cette approche n'autorise que des débits d'analyse très faibles, afin de remédier à cette limitation, plusieurs nouvelles technologies ont été développées telles que les techniques de séquençage à haut débit, les puces à ADN et les SNP-arrays. Les avantages de ces nouvelles technologies par rapport à la recherche en génomique en général, et surtout en pharmacogénétique, sont la rapidité des tests, leur cout moins cher, leur grande sensibilité et essentiellement leur fiabilité.

## Conclusion

La pharmacogénétique étudie les mécanismes d'origine génétique impliqués dans la variabilité interindividuelle de la réponse pharmacologique ou toxicologique des médicaments; dans l'espoir de déterminer les différents types de polymorphisme génétique affectant les patients marocains afin de leur fournir une prise en charge adaptée à chaque patient selon son profil. Des efforts énormes sont à déployer dans le domaine de la recherche et de la sensibilisation pour prescrire les tests de pharmacogénétiques chaque fois qu'il y a le risque de toxicité ou de non-réponse du traitement.
